# Oncolytic herpes simplex virus-based strategies: toward a breakthrough in glioblastoma therapy

**DOI:** 10.3389/fmicb.2014.00303

**Published:** 2014-06-20

**Authors:** Jianfang Ning, Hiroaki Wakimoto

**Affiliations:** Department of Neurosurgery, Brain Tumor Research Center, Massachusetts General Hospital, Harvard Medical SchoolBoston, MA, USA

**Keywords:** glioblastoma, oncolytic virus, herpes simplex virus type 1, gene therapy, glioblastoma stem cells, combination therapy, synergy, molecular targeted therapy

## Abstract

Oncolytic viruses (OV) are a class of antitumor agents that selectively kill tumor cells while sparing normal cells. Oncolytic herpes simplex virus (oHSV) has been investigated in clinical trials for patients with the malignant brain tumor glioblastoma for more than a decade. These clinical studies have shown the safety of oHSV administration to the human brain, however, therapeutic efficacy of oHSV as a single treatment remains unsatisfactory. Factors that could hamper the anti-glioblastoma efficacy of oHSV include: attenuated potency of oHSV due to deletion or mutation of viral genes involved in virulence, restricting viral replication and spread within the tumor; suboptimal oHSV delivery associated with intratumoral injection; virus infection-induced inflammatory and cellular immune responses which could inhibit oHSV replication and promote its clearance; lack of effective incorporation of oHSV into standard-of-care, and poor knowledge about the ability of oHSV to target glioblastoma stem cells (GSCs). In an attempt to address these issues, recent research efforts have been directed at: (1) design of new engineered viruses to enhance potency, (2) better understanding of the role of the cellular immunity elicited by oHSV infection of tumors, (3) combinatorial strategies with different antitumor agents with a mechanistic rationale, (4) “armed” viruses expressing therapeutic transgenes, (5) use of GSC-derived models in oHSV evaluation, and (6) combinations of these. In this review, we will describe the current status of oHSV clinical trials for glioblastoma, and discuss recent research advances and future directions toward successful oHSV-based therapy of glioblastoma.

## Introduction

Glioblastoma (GBM) is the most common and aggressive primary brain tumor in adults (Wen and Kesari, 2008). The current standard treatment for GBM consists of surgical resection followed by combination of radiotherapy and chemotherapy with the alkylating agent temozolomide (TMZ). Despite this multimodality approach, tumors inevitably recur for which therapeutic options are limited. Median survival of GBM patients is only 14.6 months (Stupp et al., 2005) and this remarkably poor outcome has not improved substantially over the last three decades. Therapeutic challenges include the invasive and infiltrative nature of GBM growth that makes surgical resection always incomplete, and the resistance of tumor cells to the conventional cytocidal therapies. Molecular targeted agents have not achieved an increase in overall survival (Chinot et al., 2014; Gilbert et al., 2014). Thus, there is an urgent need to develop novel, effective therapies for this devastating malignancy.

Oncolytic viruses (OV) have been drawing a great deal of attention as biological anti-cancer agents, with a distinct mechanism of action from conventional cancer therapeutics. OV is a replication competent virus, either genetically engineered or naturally occurring, which selectively replicates in tumor cells and causes tumor cell death (Russell et al., 2012). The production of virus progeny leads to secondary infection and virus spread within the tumor and eventual tumor destruction. This process, however, proceeds while sparing normal cells. Selective replication of OV in tumor cells can be achieved by taking advantage of aberrations found in cancer cells, which include defective innate anti-viral and apoptotic response to viral infection. Thus, viral genes that are not essential for viral growth but required for viral propagation in normal cells can be mutated or deleted to engender tumor selectivity. Many viruses, including both DNA [e.g., herpes simplex virus (HSV)] and RNA (e.g., poliovirus) viruses, have been studied as potential OV platforms for cancer therapeutics.

For GBM treatment, HSV, adenovirus (Chiocca et al., 2004), poliovirus (Gromeier et al., 2000; Goetz and Gromeier, 2010), Newcastle disease virus (Freeman et al., 2006), reovirus (Forsyth et al., 2008; Kicielinski et al., 2014), parvovirus (Herrero et al., 2004), and measles virus (Allen et al., 2013) are at different stages of clinical OV development after preclinical investigations showing anti-GBM activities (Wollmann et al., 2012; Murphy and Rabkin, 2013). Oncolytic HSV (oHSV) is considered particularly suited and promising for treating GBM. A double stranded DNA virus containing ~80 viral genes, HSV type 1 is a human pathogen causing illnesses such as encephalitis, therefore use of HSV as a cancer therapeutic requires genetic engineering. Historically, oHSV has undergone several generations of genetic manipulation during its development as an antitumor agent. The first engineered HSV capable of selective replication and killing GBM was reported by Martuza et al. (1991), which opened the door to applying “virotherapy” for GBM. This seminal work featured an engineered oHSV mutant, dlsptk, which lacks the viral gene encoding thymidine kinase (Gordon et al., 1983; Martuza et al., 1991). Neuropathogenicity issues, however, remained with these HSV-TK mutants. The second gene used to reduce neurovirulence and confer tumor specificity was γ34.5, and the deletion of the both copies of γ34.5 has been commonly employed to create oHSVs. The gene product of γ34.5, ICP34.5, recruits protein phosphatase 1 (PP1) to dephosphorylate eIF2α leading to inhibition of the protein synthesis shut-off caused by phosphorylated eIF2α (p-eIF2α), a crucial anti-viral defense mechanism mediated by host RNA-induced protein kinase R (PKR) (He et al., 1997). ICP34.5 also binds to the mammalian autophagy protein Beclin 1 to antagonize host autophagy responses (Orvedahl et al., 2007), and forms a complex with proliferating cell nuclear antigen (PCNA), a protein involved in DNA replication and repair (Brown et al., 1997). HSV mutants lacking ICP34.5 maintain the capability of replication in tumor cells where ICP34.5 is not required for production of infectious progeny, whereas they fail to replicate in normal neurons where ICP34.5 is essential for viral replication (MacLean et al., 1991; McKie et al., 1996, 1716). The third viral gene used to attenuate oHSV is *UL39*, which encodes the large subunit of ribonucleotide reductase (ICP6) (Goldstein and Weller, 1988; Mineta et al., 1994). ICP6 is required for efficient viral growth in non-dividing cells but not in many dividing cells with abundant dNTP pools or in cells with mutated p16 (Aghi et al., 2008). The second generation of oHSV was developed based upon the first generation mutants and contains multiple gene mutations/deletions. G207 has deletions at both γ34.5 loci and a *lacZ* gene insertion inactivating the ICP6 gene (UL39) (Mineta et al., 1995). Some newer oHSVs with additional properties have been developed based on the mutants indicated here and include so-called “armed” oHSV carrying therapeutic transgenes.

oHSV has been shown to be effective for the treatment of a variety of cancer types as reviewed in (Shen and Nemunaitis, 2006; Liu et al., 2013a), and an oHSV expressing GM-CSF has demonstrated clinical efficacy in malignant melanoma trials (Senzer et al., 2009).

Progress in improving oHSV therapy for GBM has been steady but slow; it is over 20 years since the discovery of the oncolytic potential of oHSV for GBM therapy, and clinical exploratory investigations of oHSV began more than a decade ago. Preclinical and clinical research of oHSV has not only shown great potential for GBM treatment, but also revealed drawbacks and limitations of the exiting oHSV strategies and the need for further refinement. In this article, we will summarize the current status of oHSV in GBM clinical trials, discuss recently described oHSV strategies, and propose future directions for successful oHSV application to clinical GBM therapy.

## oHSV clinical trials for GBM

HSV1716 is one of the first generation engineered oHSVs, with deletions in both copies of the neurovirulence gene γ34.5 for selective replication in tumor cells. A series of 3 phase I clinical trials using HSV1716 were conducted in the UK and reported on from 2000 to 2004 (Rampling et al., 2000; Papanastassiou et al., 2002; Harrow et al., 2004). The first study evaluated the safety of intratumoral injection of HSV1716 in 9 patients with recurrent malignant glioma (Rampling et al., 2000). HSV1716 at doses of 10^3^–10^5^ pfu (plaque forming unit) did not induce adverse clinical symptoms or reactivation of latent HSV, demonstrating the safety and feasibility of intratumoral administration of HSV1716. In the second study, 12 patients with malignant glioma (including 11 GBM) received an intratumoral injection of 10^5^ pfu HSV1716, and 4–9 days after inoculation, tumors were removed and assayed for viral replication (Papanastassiou et al., 2002). HSV1716, in excess of the input dose, was recovered from the injection site in 2 patients. PCR detected HSV DNA at the sites of inoculation in 10 patients and at distal tumor sites in 4; HSV-specific antigen was detected in 2 tumors. Five patients, including 2 sero-negative cases, showed increases in levels of HSV-specific IgG and IgM, indicating an immunological response to HSV1716. This study for the first time demonstrated that oHSV replicates in malignant gliomas without causing toxicity in both HSV-seropositive and -seronegative patients. The third trial assessed the safety of HSV1716 injection into the brain adjacent to tumor and the ability of the virus to eliminate any residual tumor (Harrow et al., 2004). Twelve patients with recurrent or newly diagnosed high-grade gliomas underwent tumor resection, and received a total of 10^5^ pfu HSV1716 injected into 8 to 10 sites around the resulting tumor cavity. There was no clinical evidence of toxicity associated with virus administration. Three patients remained alive and clinically stable at 15–22 months post-surgery and HSV1716 injection, including 1 patient in which magnetic resonance imaging (MRI) demonstrated a reduction of tumor volume over 22 months. This study showed the safety of HSV1716 injection into the brain adjacent to excised tumor, and potential efficacy of HSV1716 in glioma patients.

Results of clinical trials using G207 conducted in the US were published in 2000, 2009, and 2014 (Markert et al., 2000, 2009, 2014). A phase 1 trial designed to determine the safety of G207 inoculation into gliomas recruited 21 patients with recurrent malignant gliomas (16 GBMs and 5 anaplastic astrocytomas), and used escalating vector dose from 1 × 10^6^ to 3 × 10^9^ pfu (Markert et al., 2000). No serious toxicity attributable to G207 was observed. There were radiographic (MRI) and neurological findings suggestive of anti-tumor activity. In 4 patients who underwent tumor re-resection after G207 inoculation, PCR detected G207 DNA in tumors from 2 patients, whose resections were done 56 and 157 days after inoculation. One GBM patient who showed tumor regression in post-treatment MRI passed away due to a stroke 10 months after G207 treatment and autopsy showed no evidence of GBM.

The following phase 1b study used two inoculations of G207 before and after tumor resection in six patients with recurrent GBM (Markert et al., 2009). First virus inoculation (1.5 × 10^8^ pfu) was given intratumorally via a catheter, and 2–5 days later second virus (1 × 10^9^ pfu) was injected into the brain surrounding the resection cavity immediately following resection. One patient experienced transient hyperthermia and decreased responsiveness, which was probably related to G207 administration into the ventricles. The small sample size rendered efficacy assessment difficult, but there was no radiographic complete or partial response and median survival after G207 administration was 6.6 (2–20.75) months. Viral replication was detected in 3 patients by RT-PCR and in 5 patients by immunohistochemistry for the late protein gC. However, infectious G207 was isolated from the tumor of only one patient. No shedding of G207 was detected from serum, conjunctive and saliva. Increases in mononuclear CD3, CD8 and HAM56+ immune cell infiltrates were noted in post-G207 tissue in the majority of patients. This study demonstrated the viral replication of G207, and possible antitumor activity and an overall good safety profile of multiple dose delivery of G207, including direct inoculation into the brain adjacent to tumor resection cavities.

Very recently, the results of a phase 1 trial combining G207 with radiation therapy (NCT00157703) were reported (Markert et al., 2014). Nine recurrent glioma patients (7 GBMs) received stereotactic intratumoral inoculations of G207 at 10^9^ pfu, and 24 h later were treated with single focal irradiation (5 Gy). Treatment was well tolerated. Three patients showed marked radiographic responses, and the median survival time from G207 injection until death was 7.5 months. This study showed the safety and the potential for clinical response of the combinatorial treatment of G207 and radiation for malignant glioma.

In these clinical trials, the maximum tolerated dose (MTD) was never reached and the use of these mutant HSVs in human brain was generally well tolerated. No patient has ever developed HSV encephalitis. While efficacy evaluation was not a primary objective of these studies, instances of tumor regression by MRI or autopsy, and of long-term survival as long as 22 months after HSV1716 injection are encouraging signs (Harrow et al., 2004). Tumor resection after viral inoculation provided evidence for replication of HSV1716 (Papanastassiou et al., 2002) and G207 (Markert et al., 2009), but at relatively low frequency with 2 of 12 patients (Papanastassiou et al., 2002) and 3 of 6 patients (Markert et al., 2009), respectively. This low rate of replication detection may indicate ineffective replication and spread of oHSV, which could directly compromise its efficacy in GBM therapy. These clinical studies also showed that oHSV injection in the human brain induces immune responses, including sero-conversion and recruitment of immune cells to the site of oHSV infection (Papanastassiou et al., 2002; Harrow et al., 2004; Markert et al., 2009). Mononuclear immune cells such as macrophages and T lymphocytes could play major and different roles in modulating oHSV efficacy by prematurely clearing oHSV as innate and adaptive immune response or by attacking tumor cells as anti-tumor effectors. In this regard, the information available from these published studies is preliminary and limited, and future investigations should decipher the complex interaction of the host immune system and oHSV infection of GBM. Thus, these clinical trials, although exhibiting promise, revealed challenges and unanswered questions.

In addition to these published trials, ongoing or unpublished clinical studies testing newer oHSVs for malignant gliomas are listed in Table 1. G47Δ is a third generation oHSV created from G207 with an additional deletion within the nonessential α*47* gene. The absence of α*47* leads to increased MHC class I expression, and early expression of Us11 driven by the immediate-early α*47* promoter enhances the growth of γ34.5 mutants (Todo et al., 2001). rQNestin34.5, a HSV mutant in which one copy of the γ34.5 gene was reinserted into the UL39-deleted, γ34.5-deleted viral genome under control of the nestin promoter, exhibits increased replication in GBM cells (Kambara et al., 2005). M032 has a similar construction as M002, lacking both copies of the γ34.5 gene and expressing human IL-12 (Roth et al., 2014). All of these have shown promising safety or antitumor efficacy in preclinical GBM studies, and are currently in active clinical trials or in preparation for trials.

**Table 1:** **Oncolytic herpes simplex viruses in completed and ongoing clinical trials for malignant glioma**.

**Virus (stain)**	**Country**	Year^*^	**Status**	**Phase**	**Protocol**	**Result**	**References**
1716	UK	1996	Closed	I	Intatumoral, 10^3^–10^5^ pfu, single injection	Of 9 patients treated, 4 were alive and well 14–24 months after 1716 administration. No toxicity or serious adverse events.	Trial ID: UK0033^**^ (Rampling et al., 2000)
1716	UK	2000	Closed	I	Intatumoral, 10^5^ pfu, single injection	No toxicity or serious adverse events	(Papanastassiou et al., 2002)
1716	UK	2001	Closed	I	Adjacent brain, 10^5^ pfu, single injection	3 out of 12 patients remained alive and clinically stable at 15–22 months post-surgery and 1716. No toxicity or serious adverse events.	(Harrow et al., 2004)
1716	UK	2006	Open	II/III	Intratumoral	Unpublished	UK0136^**^
1716	USA	2013	Open	I	Intratumoral/Peritumoral	Unpublished	Trial ID: NCT02031965
G207	USA	1998	Open	I	Intratumoral, 10^6^ – 3 × 10^9^ pfu, single injection	8 out of 21 patients with decreased tumor volume. No toxicity or serious adverse events.	Trial ID: US0235^**^ (Markert et al., 2000)
G207	USA	2002	Closed	Ib	Intratumoral and adjacent brain, total 1.15 × 10^9^ pfu, two injections before and after resection.	Adverse events in 1 of 6 patients.	Trial ID: NCT00028158 (Markert et al., 2009)
G207	USA	2005	Closed	I	Intratumoral injections at 10^9^ pfu, followed by a single focal irradiation (5 Gy) 24 h later.	Marked radiographic response in 3 of 9 patients, with 7.5 months of median survival. No toxicity or serious adverse events.	Trial ID: NCT00157703 (Markert et al., 2014)
G47Δ	Japan	2009	Open	I/II	Stereotactic intratumoral injections, 2nd injection follows 5–14 days later.	Unpublished	Trial No.: UMIN000002661
rQNestin –34.5	USA	2011	Open	I	Peritumoral	Unpublished	Trial ID: US1100^**^
M032	USA	2014	Open	I	Intratumoral, single injection	Unpublished	Trial ID: NCT02062827

* Year approved or initiated.

** According to The Journal of Gene Medicine Clinical Trial site (http://www.abedia.com/wiley/index.html).

## Current strategies using oHSV for GBM therapy

Since clinical trials have shown apparently limited efficacy of oHSV for GBM, how to improve efficacy while retaining safety has become the focus of preclinical and clinical oHSV research. To this end, recent research efforts have been directed at different areas that include: (1) use of GBM cancer stem cell-derived models in oHSV evaluation, (2) design of new engineered viruses to enhance potency, (3) better understanding of the role of the cellular immunity elicited by oHSV infection of tumors, (4) combinatorial strategies with different antitumor agents with a mechanistic rationale, and (5) “armed” viruses expressing therapeutic transgenes. In this section, we will overview recent relevant publications and summarize cutting-edge strategies and advances toward improving oHSV therapy of GBM.

### Use of GBM stem cell-derived model in oHSV evaluation

With increasing knowledge of GBM biology, the propagation of GBM is thought to be sustained and driven by a subpopulation of neoplastic cells. These cells, termed GBM stem cells (GSCs), exhibit stem cell-like properties of self-renewal and multi-potential differentiation, and efficiently generate tumors upon implantation to immunocompromised mice (Nduom et al., 2012). GSCs appear to play a key role in tumor invasion, resistance to various treatments, and recurrence (Eyler and Rich, 2008) and thus are considered a critical therapeutic target.

We investigated the efficacy of oHSV with different genetic mutations against GSCs isolated from surgical specimens of human GBMs (Wakimoto et al., 2009). Although UL39 (ICP6) mutant FΔ6 was as efficacious as wild type strain F, γ34.5-deleted oHSV such as R3616 and G207 were unable to replicate in GSCs, exhibiting an attenuated phenotype. However, additional deletion of α47 reversed this phenotype, as G47Δ was able to replicate, spread, and kill both CD133+ and CD133– cells, as well as repress secondary sphere formation of GSCs. Thus, potency of oHSV against human GSCs correlated with oHSV mutations. We also showed that a single intratumoral inoculation of G47Δ prolonged survival of immunocompromised mice harboring highly invasive GSC-derived intracranial GBM. Friedman et al also showed that both CD133+ and CD133− GBM cells are targetable by oHSV, and that expression of CD111 (nectin-1), an HSV entry receptor, is an important factor determining sensitivity to oHSV (Friedman et al., 2009).

GBM contains abundant hypoxic regions that promote the GSC-like phenotype and confer resistance to chemo- and radiotherapy (Li et al., 2009; Seidel et al., 2010). Hypoxia was shown to enhance replication of oHSV G207 in U87 GBM xenografts *in vitro* and *in vivo* (Aghi et al., 2009). Sgubin et al. examined the therapeutic effects of G47Δ in patient-derived GSCs under normoxia and hypoxia (Sgubin et al., 2012). G47Δ infection of GSCs was able to counteract hypoxia-mediated enhancement of the stem-like properties of GSCs, inhibiting their self-renewal and stem cell marker CD133 expression. In orthotopic human GSC xenografts, inoculation of a G47Δ derivative, G47ΔUs11fluc, demonstrated an equivalent frequency of viral infection and replication in hypoxic and nonhypoxic tumor areas, highlighting the ability of oHSV G47Δ to target GSCs in a hypoxic niche environment (Sgubin et al., 2012). On the other hand, γ34.5-deleted oHSV C101 was shown to exhibit reduced replication and/or cytotoxicity against GSC-like human xenograft lines under hypoxic as opposed to normoxic conditions despite increased CD111 (nectin-1) expression mediated by hypoxia (Friedman et al., 2012).

Unlike human GBM xenografts, syngeneic GBM models in immunocompetent mice allow critical evaluation of how oHSV-induced adaptive immune responses impact on oHSV therapy of GBM. Murine 005 GBM cells, possessing heterozygous *Tp53* and activated Ras and Akt, exhibit stem-like properties, including high-level expression of mProminin-1 (CD133 equivalent), and demonstrate histopathological hallmarks of GBM such as cellular heterogeneity, invasion and hypervascularity (Marumoto et al., 2009). This syngeneic GSC model enabled detailed characterization of the multifaceted efficacy of G47Δ-mIL12, including assessment of direct oncolysis, regulatory T cell downregulation, and anti-angiogenesis (Cheema et al., 2013).

### Design of new engineered viruses to enhance potency

Although oHSVs that lack γ34.5 are capable of replication in tumor cells, the clinical trials testing HSV1716 and G207 suggested that these γ34.5-null attenuated oHSVs may not be potent enough to provide substantial efficacy for GBM. Preclinical research has continued to seek new designs of oHSV that could achieve both potency and safety. Such approaches include deletion of viral genes with partially or fully intact γ34.5 and receptor-mediated tumor specific targeting.

#### The use of viral gene deletion

Us3 encodes a serine-threonine kinase that inhibits virus-induced apoptosis and Akt activation. As apoptotic pathways are frequently dysfunctional in tumor cells, oHSV with a Us3 deletion replicates preferentially in tumor cells, but induces apoptosis in normal cells, preventing further replication (Liu et al., 2007). However, Us3 mutant R7041 is not safe enough for intracerebral inoculation. A novel oHSV, MG18L, was constructed that contains a US3 deletion and an inactivating LacZ insertion in UL39 (Kanai et al., 2011). MG18L was severely neuroattenuated in mice, replicates well, and activates Akt in human GSCs. *In vivo*, intratumoral injection of MG18L had anti-GBM activity in an orthotopic GSC xenograft model (Kanai et al., 2011). Importantly, MG18L like its parental Us3 mutant R7041 was synergistic with inhibitors of phosphatidylinositol 3-kinase (PI3K) in killing tumor cells, and this will be discussed later.

The γ34.5 gene of HSV plays a central role in neuropathogenicity and is deleted for tumor selectivity in thus far all oHSVs clinically evaluated for treating malignant gliomas (HSV1716 and G207). Unfortunately, deletion of γ34.5 attenuates virus replication even in cancer cells, especially human GSCs (Wakimoto et al., 2009). γ34.5-encoded ICP34.5 is a multifaceted protein, and one of its diverse functions is to interfere with autophagy by binding to a cellular protein, Beclin 1 (Atg6) (Orvedahl et al., 2007). Kanai et al. developed a new oHSV, Δ68H-6, with deletion of the γ34.5 Beclin 1 binding domain (BBD, aa 68–87), rather than the whole γ34.5, in addition to UL39 (ICP6) inactivation (Kanai et al., 2012b). Δ68H-6 exhibited minimal neuropathogenicity in HSV-susceptible mice as opposed to single mutants for BBD (Δ68H) and UL39 (Δ68HR-6) (Kanai et al., 2012b). Δ68H-6 replicated well in human glioma cell lines and GSCs, effectively killing these cells *in vitro* and prolonging survival of athymic mice bearing orthotopic human U87 and GSC-derived tumors (Kanai et al., 2012b). In contrast, γ34.5 deleted 1716 and 1716-6 barely replicated in GSCs. As expected, infection of Δ68H-6 or 1716-6 induced autophagy in glioma cells, but inhibition of autophagy had no effect on virus replication or p-eIF2α levels (Kanai et al., 2012b).

#### Receptor mediated targeting of tumor cells

*EGFR* gene amplification is found in 40% of GBMs and is associated with overexpression of epidermal growth factor receptor (EGFR) and its truncated mutant form, EGFRvIII (Sugawa et al., 1990; Ekstrand et al., 1992). EGFRvIII presents a tumor specific epitope on the cell surface and can be specifically recognized by a single chain antibody, MR1-1. An oHSV was engineered by fusing MR1-1 to the viral glycoprotein gC, an envelope protein involved in initial virus binding to heparin sulfate on the cell surface (Grandi et al., 2010). This mutant oHSV had 5-fold increased infectivity for EGFRvIII-bearing U87 glioma cells compared to mutant receptor-deficient cells. *In vivo*, the MR1-1-modified-gC oHSV imparted sustained infection of EGFRvIII^+^ U87 glioma subcutaneous xenografts (Grandi et al., 2010). Using a similar strategy, another EGFR-retargeted oHSV was created by introducing a scFv antibody for human EGFR into a mutant form of viral glycoprotein gD (Uchida et al., 2013). The retargeted oHSV, after having another entry accelerating mutation in gB, efficiently and exclusively entered cells that express EGFR. This oHSV, KNE, demonstrated safety upon intracerebral inoculation in mice and increased survival in an orthotopic mouse model of human GBM with up to 73% of animals showing complete response (Uchida et al., 2013).

R-LM113 is a recombinant HSV created by inserting a scFv antibody against HER2, human epidermal growth factor 2, into the gD gene (Menotti et al., 2008). R-LM113 is fully retargeted to HER2 since this recombinant HSV lost the ability to enter cells through the natural gD receptors, HVEM and nectin-1, but could selectively infect HER2-expressing cells. In vivo, R-LM113 was safe upon inoculation to the brains of HSV-permissive mice, and was efficacious against PDGF-induced malignant glioma as the treatment improved survival of brain tumor-bearing NOD/SCID and immunocompetent BALB/c mice (Gambini et al., 2012; Reisoli et al., 2012). Thus, tropism manipulation through retargeting fully replication competent HSV to GBM-associated receptors might provide an avenue for increased infectivity and specificity for the target cell as well as efficacy, without compromising safety.

#### Understanding virus and host interaction

Elucidation of tumor host responses to HSV and oncogenic pathways that modulate virus replication could lead to the discovery of oHSV efficacy biomarkers and design of novel oHSVs. Recently, activated STAT3 signaling, which represents a central hub in malignant glioma progression and maintenance, was shown to enhance rQNestin34.5 replication (Okemoto et al., 2013b). This is likely due to STAT3 inhibition of type 1 interferon responses in infected glioma cells (Okemoto et al., 2013b). Thus, GBM with elevated STAT3 may represent a preferable target for oHSV therapy.

### Better understanding of the role of the cellular immunity elicited by oHSV infection of tumors

Host immune responses to oHSV have been shown to be a barrier to efficient viral replication and spread within tumor after initial infection, which could compromise oHSV therapy for GBM (Ikeda et al., 1999; Wakimoto et al., 2004; Fulci et al., 2007). However, the role of different immune cell types and molecular mechanisms regulating oHSV infection has not been fully elucidated. Alvarez-Breckenridge et al. showed that oHSV infection induced rapid recruitment and activation of natural killer (NK) cells, which led to premature viral clearance and hindrance to oHSV anti-tumor efficacy in both athymic and immunocompetent mouse models (Alvarez-Breckenridge et al., 2012b). Human NK cells preferentially killed oHSV-infected GBM cells through the interaction between natural cytotoxicity receptors (NCR) NKp30 and NKp46 on NK cells and their ligands on infected GBM cells (Alvarez-Breckenridge et al., 2012b). oHSV titers and efficacy were increased in GBM in Ncr1^−/−^ mice. Therefore, oHSV therapy of GBM is partly limited by an antiviral NK cell response involving specific NCRs, uncovering novel potential targets to enhance oHSV cancer therapy. The same group led by Dr. Chiocca further demonstrated that NK cell action against oHSV-infected GBM cells was lessened by the histone deacetylase inhibitor valproic acid through inhibition of STAT5/T-BET signaling and generation of γ-interferon (Alvarez-Breckenridge et al., 2012a). Given that valproic acid is being used in the clinic as an anti-convulsant, this observation is significant in opening up an opportunity for pharmacological modification of the innate immune response to oHSV to enhance virus replication and efficacy in humans.

The cellular immune response to oHSV is a complex phenomenon involving innate immunity to oHSV that is likely deleterious to efficacy and the subsequent adaptive anti-tumor response that is beneficial. Indeed oHSV infection of tumors acts as an *in situ* vaccination to elicit tumor specific T lymphocytes effector responses (Toda et al., 1999). Such an approach to inducing anti-tumor immunity by oHSV can be further boosted by taking advantage of oHSV as a gene delivery vector and expressing immune enhancing factors. Two recent publications described new armed oHSVs expressing murine fms-like tyrosine kinase 3 ligand (Flt3L) and IL12 (Barnard et al., 2012; Cheema et al., 2013), and showed their efficacy to be superior to control oHSV in syngeneic mouse GBM models, underscoring the utility of the immune activating strategy. These strategies will be discussed later in the “armed oHSV” section.

### Combinational strategies with different antitumor agents with a mechanistic rationale

The history of cancer therapy development has demonstrated that combinations of multiple anti-cancer modalities are often more efficacious than each single treatment for refractory cancer. Successful targeting of GBM, with its heterogeneity, invasiveness, and multiple oncogenic pathways, likely requires such multi-modal approaches. Recent research has uncovered promising combinatorial approaches employing oHSV and other agents that are mechanism-based and often exhibit synergistic anti-cancer effects (Kanai and Rabkin, 2013).

#### Combination with conventional therapy

How to incorporate oHSV into the existing standard of care for GBM is a practical and important issue from the perspective of oHSV translation to the clinic. Currently used radiation and TMZ chemotherapy are both genotoxic, while HSV infection also elicits cellular DNA damage responses to facilitate viral replication (Taylor and Knipe, 2004; Lilley et al., 2005; Chaurushiya and Weitzman, 2009). This implies that DNA damage inducers currently used for GBM and oHSV can modulate common DNA repair signaling and provide synergistic impact on cell death.

Previously, TMZ-induced tumor-protective DNA repair pathways, involving cellular GADD34 and ribonucleotide reductase, were shown to enhance oHSV G207-mediated oncolysis in glioma cells (Aghi et al., 2006). Subsequently, Kanai et al reported that G47Δ acts synergistically with TMZ in killing human GSCs through oHSV-mediated manipulation of DNA damage responses (Kanai et al., 2012a). Mechanistically, activated ataxia telangiectasia mutated (ATM) was found to be a crucial mediator of synergy. Upon G47Δ infection, activated ATM relocalized to HSV DNA replication compartments where it likely enhanced oHSV replication but could not participate in repairing TMZ-induced DNA damage, resulting in extensive DNA damage and cell death. For TMZ-resistant GBM cells expressing O6-methylbenzylguanine DNA methyltransferase (MGMT), G47Δ and TMZ were still synergistic in the presence of an MGMT antagonist (Kanai et al., 2012a). Combined G47Δ and TMZ treatment induced robust DNA damage and markedly extended survival of mice bearing MGMT-negative GSC-derived intracranial tumors at TMZ doses attainable in patients. Triple combination therapy with MGMT antagonist was effective against MGMT-positive orthotopic xenografts (Kanai et al., 2012a).

G47Δ was also combined with etoposide, a topoisomerase 2 inhibitor, and the combination showed moderate synergy in killing human GSCs and GBM cell lines (Cheema et al., 2011). This combination did not enhance virus replication, but significantly increased caspase-mediated apoptosis. *In vivo*, the combined treatment of a single cycle of low-dose etoposide with intratumoral G47Δ injection significantly extended survival of athymic mice bearing intracranial human GSC-derived tumors over each single treatment (Cheema et al., 2011).

The combination of OV with ionizing radiation (IR) has been shown to increase therapeutic efficacy through viral interference with DNA repair following irradiation of glioma cells (Hadjipanayis and DeLuca, 2005) and enhanced viral replication by IR (Advani et al., 2006). When combined with IR, γ34.5 mutant oHSV resulted in increased viral replication and anti-tumor effects in murine glioma models (Advani et al., 1998). Advani et al. used γ34.5-deleted HSV-1 carrying a late promoter driven luciferase in U87 glioma xenografts and administered IR before or after viral injection to determine optimal temporal sequencing (Advani et al., 2011). Delivering radiation 6–9 h after oHSV injection, i.e., coinciding with the onset of late viral gene expression, resulted in the greatest luciferase expression, infectious virus production, and tumor-xenograft regression (Advani et al., 2011). Therefore, when combined with oHSV, IR administration at an optimal time during the HSV replicative cycle may maximize the combination effect.

#### Combination with molecularly targeted agents

Molecular analysis of GBMs has shown that genetic alterations in the PI3K/Akt pathway occur in about 80% of tumors (Parsons et al., 2008). These molecular changes confer GBMs with proliferative and survival advantages and thus offer an important target for GBM therapy. oHSV MG18L has a deletion of U_S_3 which results in Akt activation (phosphorylation) in infected GBM cells. Combination of MG18L and PI3K or Akt inhibitors (e.g., LY294002, GDC-0941 and Triciribine) was synergistic in killing GSCs and glioma cell lines, but not human astrocytes, through enhanced induction of apoptosis (Kanai et al., 2011). *In vivo*, the combination significantly prolonged survival of mice bearing orthotopic GBM xenografts, as compared to either agent alone, through enhanced induction of apoptosis (Kanai et al., 2011). This is a good example of a mechanism-based combination strategy that exploits oHSV-mediated alteration of an oncogenic signaling pathway in tumor cells.

#### Combination with anti-angiogenic agents

One of the pathological hallmarks of GBM is marked neovascularity, which is considered to promote GBM progression (Das and Marsden, 2013). As an adjuvant strategy to treat GBM, anti-angiogenic therapy has been extensively investigated in preclinical and clinical studies (Gerstner and Batchelor, 2012).

An angiostatic peptide, cyclic Arg-Gly-Asp (cRGD, cilengitide), binds to and blocks integrins (Brooks et al., 1994; Friedlander et al., 1995) that are highly expressed on tumor-associated endothelial cells. Cilengitide pretreatment of immunocompetent rats bearing orthotopic GBM enhanced treatment with the UL39 mutant oHSV hrR3 (Kurozumi et al., 2007). hrR3 titer was elevated with the cilengitide pretreatment through the reduction of tumor vascular permeability, immune cell infiltration, and γ-interferon production. These observations were consistent with the finding that suppression of macrophage infiltration into brain tumors enhances oHSV amplification and spread (Fulci et al., 2007). Cilengitide was also used with a γ34.5-deleted, UL39-disrupted oHSV, rHSVQ, modified to express vasculostatin (Vstat120), the fragment of brain-specific angiogenesis inhibitor-1 (BAI1), to treat GBM (Fujii et al., 2013). *In vitro*, treatment with the armed oHSV, named RAMBO (Hardcastle et al., 2010), plus cilengitide enhanced the inhibition of endothelial tube formation and mediated synergistic cytotoxicity on glioma cells. *In vivo*, combination therapy significantly increased the survival of GBM-bearing athymic mice compared with RAMBO or cilengitide monotherapy (Fujii et al., 2013). Thus, cilengitide enhanced vasculostatin-expressing virus therapy for malignant glioma.

Zhang et al demonstrated another successful example of combining two agents with anti-angiogenic properties (Zhang et al., 2012). In athymic mice bearing intracerebral U87 GBM, systemic administration of Bevacizumab, a monoclonal antibody directed at VEGF, increased spread of G47Δ-mAngio, G47Δ oHSV engineered to secrete anti-angiogenic protein murine angiostatin, as well as the anti-angiogenic effect. Multimodal therapy using Bevacizumab plus G47Δ-mAngio increased animal survival over each monotherapy alone, which was associated with reduction of collagen and matrix metalloproteinases (MMP) 2 and 9 in tumors (Zhang et al., 2012).

#### Others

rQNestin34.5 is a genetically modified version of oHSV rHSVQ with a nestin promoter/enhancer driving expression of a single copy of γ34.5, rendering an increased ability to replicate (Kambara et al., 2005). However, this promoter becomes extensively methylated in infected glioma cells, reducing ICP34.5 expression and oncolytic potency. Okemoto al. used demethylating drugs to determine if they improve the efficacy of rQNestin34.5 therapy in malignant glioma models (Okemoto et al., 2013a). 5-Aza improved rQNestin34.5 replication and tumor cell lysis *in vitro*. *In vivo*, intratumoral injection of rQNestin34.5 mixed with demethylating agents, 5-Aza or decitabine (5-aza-2'-deoxycytidine), significantly prolonged the survival of athymic mice harboring intracranial human glioma xenografts over single agents alone. Interestingly, valproic acid also demethylated the nestin promoter and increased rQNestin34.5 replication (Okemoto et al., 2013a).

Copper in serum supports angiogenesis (Soncin et al., 1997; Hu, 1998) and inhibits replication of wild-type HSV-1 (Shishkov et al., 1997). Copper chelators are currently being investigated as an antiangiogenic and antineoplastic agent for cancer patients (Lin et al., 2013). Yoo et al showed that physiologically relevant concentrations of copper (1 mg/L) inhibits oHSV rQNestin34.5 infection and replication, and copper chelation with ATN-224 reversed this copper-mediated oHSV inhibition (Yoo et al., 2012). *In vivo*, systemic administration of ATN-224 augmented therapeutic efficacy of intratumoral rQNestin34.5 against intracranial GBM in athymic mice, which was associated with reduced vascular leakiness (Ktrans) and blood vessel density, suggesting anti-angiogenic effects by ATN-224 (Yoo et al., 2012). Importantly, systemic ATN-224 enhanced delivery and efficacy of systemically administered oHSV hrR3, as the combination significantly reduced tumor growth likely through ATN-224-mediated increased stability of oHSV in serum (Yoo et al., 2012).

### “Armed” viruses expressing therapeutic transgenes

The presence of genes not essential for virus replication allows oHSV to accommodate relatively large sizes of exogenous DNA. oHSV engineered to carry therapeutic genes, so-called “armed oHSV,” has been used to deliver and express therapeutic proteins in tumor cells in an attempt to enhance antitumor efficacy. The following sections will discuss recently described strategies in which “armed” oHSVs play a central role.

#### Immunomodulating genes

GBM promotes an immunosuppressive tumor microenvironment and the immune status of GBM patients is compromised (Rolle et al., 2012). oHSV can function as an “*in situ* vaccination” since oHSV-mediated tumor cell killing elicits anti-tumor immunity (Toda et al., 1999). Although stimulation of inflammation can lead to virus clearance before its amplification, strategies to enhance oHSV efficacy by expressing cytokines with immune activation properties appear promising.

Interleukin 12 is a prominent Th1 response inducer and known to promote anti-tumor cellular immunity. Expression of IL-12 in the context of oHSV enhances efficacy in the treatment of murine model of GBM (Hellums et al., 2005). Cheema et al. further demonstrated the utility of a murine IL12 armed oHSV, G47Δ-mIL12, in an immunocompetent murine GSC model (Cheema et al., 2013). G47Δ-mIL12 infects and replicates similarly to the unarmed oHSV counterpart *in vitro*, whereas it significantly enhances survival in syngeneic mice bearing intracerebral 005 GBMs *in vivo* (Cheema et al., 2013). Mechanistically, G47Δ-mIL12 evoked multifaceted beneficial responses including increased IFN-γ release, anti-angiogenesis, and reduction of regulatory T cell infiltration to the tumor microenvironment. *In vivo* NK cell depletion and use of athymic mice revealed the requirement of T, but not NK cells for the efficacy of G47Δ-mIL12 (Cheema et al., 2013). Using the same HSV backbone, G47Δ-Flt3L was created to stimulate anti-tumor immunity through on site expression of soluble Flt3L, a cytokine capable of differentiating hematopoietic precursors into plasmacytoid and conventional dendritic cells (DCs) and mobilizing them from bone marrow (Barnard et al., 2012). In culture, high levels of Flt3L expressed by G47Δ-Flt3L did not affect viral replication or a cytotoxic effect on glioma cells. Direct inoculation of G47Δ-Flt3L into intracerebral CT2A gliomas in C57BL/6 mice resulted in detectable levels of Flt3L in the blood and was superior to parental G47Δ in prolonging survival of animals (Barnard et al., 2012).

A study examining the safety and biodistribution of M032, a γ34.5-deleted oHSV expressing human IL-12, in HSV-susceptible *Aotus* nonhuman primates was described very recently (Roth et al., 2014). A single intracerebral inoculation of 10^6^ or 10^8^ pfu of M032 caused temporary slight body weight loss and mild to moderate inflammatory reactions in the brain, but was overall well tolerated. Viral DNA was detected in the brain and, at lower levels, in the spleen up to 91-day post inoculation (Roth et al., 2014).

#### Anti-angiogenic genes

Anti-angiogenic therapy targets neovasculature within the tumor microenvironment. A number of secreted proteins capable of inhibiting tumor-associated neo-angiogenesis have been identified, and used to arm oHSV to enhance overall efficacy through dual targeting of tumor cells and stromal vascular cells.

An oncolytic HSV (γ34.5-, ICP6-) carrying an endostatin-angiostatin fusion gene (VAE) killed GSCs and the expression of endo-angio inhibited proliferation of human brain microvascular endothelial cells (HBMEC) compared to control oHSV (Zhu et al., 2011). *In vivo*, intratumoral injection of VAE extended survival of mice harboring intracerebral human GSC xenografts compared with control monotherapies, non-transgene encoding rHSV-1 or recombinant endostatin (Zhang et al., 2014).

Angiostatin is an antiangiogenic polypeptide, while interleukin-12 (IL-12) is an immunostimulatory cytokine with anti-angiogenic effects. A unique combination strategy using oHSVs expressing angiostatin (G47Δ-mAngio) and IL-12 (G47Δ-mIL12) was tested in orthotopic human GBM xenograft models (Zhang et al., 2013). Intratumoral injection of G47Δ-mAngio and G47Δ-mIL12 together in mice bearing intracranial U87 or GSCs-derived tumors significantly prolonged survival compared to each armed oHSV alone. This was associated with increased anti-angiogenesis and virus spread, as well as decreased macrophage infiltration (Zhang et al., 2013). These data support the use of oHSVs expressing multiple anti-angiogenic factors to improve efficacy in GBM.

#### Prodrug-activating genes

Prodrug-activating gene or suicide gene therapy uses the systemic delivery of an inactive prodrug coupled with tumor-specific expression of a drug-activating enzyme (the suicide gene), which converts the nontoxic prodrug to cytotoxic metabolites and causes tumor cell death (Duarte et al., 2012). Owning to limited off-site presence of the toxic substance, this strategy can negate serious adverse effects on normal tissues associated with systemic chemotherapy. oHSV armed with prodrug-activating genes not only exerts direct oncolytic killing but also produces bystander effects mediated by cytotoxic metabolites released from oHSV-infected cells.

MGH2, derived from MGH1 containing deletions of both copies of γ34.5 and an inactivating insertion in UL39, carries two prodrug-activating transgenes: the cytochrome P450 2B1 (CYP2B1) and the secreted human intestinal carboxylesterase (shiCE) (Tyminski et al., 2005). CYP2B1 converts cyclophosphamide (CPA) into the active DNA-alkylating metabolite, phosphoramide mustard (PM), and shiCE activates irinotecan (CPT11) to the active topoisomerase 1 inhibitor, SN-38, in target cells. MGH2 exhibited anti-tumor activity against human glioma cells both *in vitro* and *in vivo*, which was enhanced by the addition of CPA and CPT11 (Tyminski et al., 2005). For clinical translation, Dr. Chiocca's group created MGH2.1 by removing the GFP expression cassette from MGH2, and performed toxicology and biodistribution studies (Kasai et al., 2013). After intracerebral injection in BALB/c mice, MGH2.1 DNA was detected in brains for up to 60 days, and expression of virally encoded genes was restricted to brain. Intracranial inoculation of MGH2.1 induced a small increase in serum IL-6 levels and did not cause lethality in HSV-permissive BALB/C mice at 10^8^ pfu without prodrugs and at 10^6^ with prodrugs. These safety and toxicology data justify a clinical trial of intratumoral injection of MGH2.1 with peripheral administration of CPA and/or CPT11 in patients with malignant gliomas.

#### Pro-apoptotic genes

Apoptotic pathways are often dysregulated in tumors, including GBM, and apoptosis induction with pro-apoptotic gene transfer has been investigated as an anti-GBM strategy (Abe et al., 2002; Lee et al., 2002). However, HSV, like other viruses, has inherent mechanisms that prevent host cells from undergoing premature apoptosis to sustain replication (Koyama and Miwa, 1997; Aubert and Blaho, 1999), and expressing exogenous apoptosis-inducing gene in the context of oHSV had not been explored till recently.

Tamura et al. created a recombinant G47Δ oHSV that bears a transgene encoding a secretable TRAIL (tumor necrosis factor-related apoptosis-inducing ligand) (Tamura et al., 2013). This oHSV-TRAIL (G47Δ-TRAIL) showed enhanced cytotoxicity against TRAIL- or oHSV-resistant GBM cells compared to control oHSV without transgene. oHSV-TRAIL downregulated extracellular signal-regulated protein kinase (ERK)-mitogen-activated protein kinase (MAPK) and upregulated c-Jun N-terminal kinase (JNK) and p38-MAPK signaling, and induced resistant GBM cells to undergo apoptosis via activation of caspase-8, -9, and -3 (Tamura et al., 2013). Furthermore, intratumoral inoculation of oHSV-TRAIL inhibited tumor invasiveness, and increased survival of SCID mice bearing oHSV-resistant intracerebral GBMs (Tamura et al., 2013), revealing the unique efficacy of G47Δ-TRAIL for treatment-refractory GBM.

## Summary and future direction

**Figure 1:**
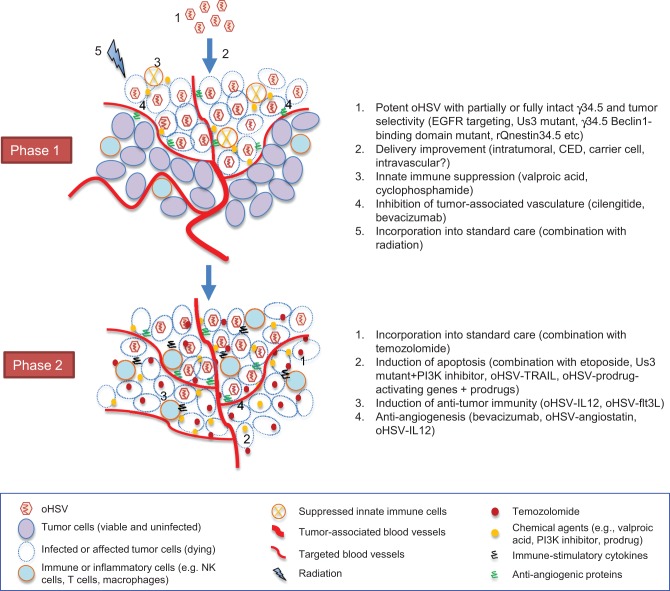
**Two-phasic process that oHSV-based strategies go through toward successful GBM tumor elimination**. For each phase, representative interventional approaches that we discussed in the text are listed on the right.

oHSV-based treatment of GBM proceeds through a dynamic, complex process that involves virus infection, replication and spread, and activation of inflammatory and immune responses as well as modulation of tumor microenvironments. As we discussed, these variable aspects of oHSV therapy provide a variety of opportunities for interventions to improve overall efficacy and maintain safety. Furthermore, oHSV-mediated activation of cellular pathways such DNA repair response and PI3K/Akt pathway can be exploited by combinatorial approaches to enhance tumor cell death. In a simplified view, the process that takes place during oHSV-based therapy can be divided into two phases (Figure 1). In phase 1, oHSV infects GBM cells, replicates, produces progeny and spreads within the tumor, resulting in oncolytic death of infected tumor cells. In subsequent phase 2, different strategies could be summoned that include activation of anti-tumor immunity and apoptosis induction in an attempt to eliminate residual unaffected part of the tumor with the goal to prevent relapse and enhance overall efficacy.

One major factor determining the success during phase 1 is delivery of therapeutic virus, which we did not discuss in detail in this article. Direct injection into tumor or brain surrounding the resection cavity is the only delivery method clinical trials have utilized. Although this approach enables oHSV to get into tumor bypassing the blood-brain barrier, there remains an issue how to deliver oHSV to tumor cells distant from the injection site. Convection-enhanced delivery (CED) (Debinski and Tatter, 2009) may enhance spatial distribution of intracerebrally injected oHSV (Hadjipanayis et al., 2008). Taking advantage of their capacity to home to tumors, neural or mesenchymal stem cells have been used as a vehicle for OV (Herrlinger et al., 2000; Ahmed et al., 2011; Duebgen et al., 2014). Virus-loaded stem cells can migrate and release virus at tumor sites distant from the injection. In addition, cell-carried viruses appear to be protected from clearance by cellular immunity. Intravascular delivery, either systemic intravenous or via carotid arterial, have been attempted for oHSV (Ikeda et al., 1999; Shikano et al., 2011) and other viruses (Liu et al., 2013b), and this approach may be worth revisiting with the aid of compounds such as CPA, valproic acid or copper chelator.

As summarized in Figure 1, we have now gained substantive knowledge about an array of strategies that could be useful to improve oHSV efficacy for GBM in the clinic. It is possible that achieving both efficient oHSV distribution within tumor (phase 1) and induction of extensive death of cells including oHSV-uninfected cells (phase 2) is necessary to obtain substantial efficacy. This could entail combined use of distinct and diverse approaches that include more potent but tumor-selective virus, current standard of care, anti-angiogenic agents, innate immune suppressors or molecular targeted agents, and on-site expression of therapeutic transgenes. The sequence and timing of each treatment may need careful consideration to optimize impacts. Because numerous combinations are now available, how to design feasible and mechanistically reasonable protocols aimed at efficient assessment of both safety and efficacy has become a significant challenge in clinical translation of novel oHSV strategies.

### Conflict of interest statement

The authors declare that the research was conducted in the absence of any commercial or financial relationships that could be construed as a potential conflict of interest.
